# *Acanthamoeba* containing endosymbiotic chlamydia isolated from hospital environments and its potential role in inflammatory exacerbation

**DOI:** 10.1186/s12866-016-0906-1

**Published:** 2016-12-15

**Authors:** Tatsuya Fukumoto, Junji Matsuo, Torahiko Okubo, Shinji Nakamura, Kentaro Miyamoto, Kentaro Oka, Motomichi Takahashi, Kouji Akizawa, Hitoshi Shibuya, Chikara Shimizu, Hiroyuki Yamaguchi

**Affiliations:** 1 Hokkaido University Hospital, Nishi-5 Kita-14 Jo, Kita-ku, Sapporo, Hokkaido 060-8648 Japan; 2 Department of Medical Laboratory Science, Faculty of Health Sciences, Hokkaido University Graduate School of Health Sciences, Nishi-5 Kita-12 Jo, Kita-ku, Sapporo, Hokkaido 060-0812 Japan; 3 Division of Biomedical Imaging Research, Juntendo University Graduate School of Medicine, 2-1-1 Hongo, Bunkyo-ku, Tokyo, 113-8421 Japan; 4 Miyarisan Pharmaceutical Co., Ltd., 2-3-13-209, Minami, Wako-shi, Saitama 351-0104 Japan

**Keywords:** *Acanthamoeba*, Environmental chlamydiae, Hospital, IL-8, Genome sequence

## Abstract

**Background:**

Environmental chlamydiae belonging to the *Parachlamydiaceae* are obligate intracellular bacteria that infect *Acanthamoeba*, a free-living amoeba, and are a risk for hospital-acquired pneumonia. However, whether amoebae harboring environmental chlamydiae actually survive in hospital environments is unknown. We therefore isolated living amoebae with symbiotic chlamydiae from hospital environments.

**Results:**

One hundred smear samples were collected from Hokkaido University Hospital, Sapporo, Japan; 50 in winter (February to March, 2012) and 50 in summer (August, 2012), and used for the study. Acanthamoebae were isolated from the smear samples, and endosymbiotic chlamydial traits were assessed by infectivity, cytokine induction, and draft genomic analysis. From these, 23 amoebae were enriched on agar plates spread with heat-killed *Escherichia coli*. Amoeba prevalence was greater in the summer-collected samples (15/30, 50%) than those of the winter season (8/30, 26.7%), possibly indicating a seasonal variation (*p* = 0.096). Morphological assessment of cysts revealed 21 amoebae (21/23, 91%) to be *Acanthamoeba*, and cultures in PYG medium were established for 11 of these amoebae. Three amoebae contained environmental chlamydiae; however, only one amoeba (*Acanthamoeba* T4) with an environmental chlamydia (*Protochlamydia* W-9) was shown the infectious ability to *Acanthamoeba* C3 (reference amoebae). While *Protochlamydia* W-9 could infect C3 amoeba, it failed to replicate in immortal human epithelial, although exposure of HEp-2 cells to living bacteria induced the proinflammatory cytokine, IL-8. Comparative genome analysis with KEGG revealed similar genomic features compared with other *Protochlamydia* genomes (UWE25 and R18), except for a lack of genes encoding the type IV secretion system. Interestingly, resistance genes associated with several antibiotics and toxic compounds were identified.

**Conclusion:**

These findings are the first demonstration of the distribution in a hospital of a living *Acanthamoeba* carrying an endosymbiotic chlamydial pathogen.

**Electronic supplementary material:**

The online version of this article (doi:10.1186/s12866-016-0906-1) contains supplementary material, which is available to authorized users.

## Background

Obligate intracellular environmental chlamydiae belong to the *Parachlamydiaceae* (*Parachlamydia*, *Protochlamydia*, and *Neochlamydia*) [1], diverged from pathogenic chlamydiae (e.g. *Chlamydia trachomatis* or *C. pneumoniae*) 0.7–1.4 billion years ago [2]. Similar to pathogenic chlamydiae, the environmental chlamydiae have a unique developmental cycle, consisting of two distinct forms: the elementary body, an infectious form, and the reticulate body, a replicative form [3]. Through this cycle, environmental chlamydiae can grow and survive within *Acanthamoeba*, a free-living amoeba that inhabits a wide range of natural environments, such as rivers and soil [4]. Because people can unconsciously carry soil, for example on shoes, amoebae may be widely distributed in public spaces, such as hospitals.

Interestingly, recent studies have revealed the presence of environmental chlamydial DNA in mononuclear cells of sputa or bronchoalveolar lavage samples from patients with bronchitis [5–7]. It is possible that environmental chlamydiae (*Parachlamydia* or *Protochlamydia*) can cause inhalation pneumonia, for example hospital-acquired pneumonia in immune compromised hosts, such as HIV-infected patients or organ transplant recipients [8]. Thus, environmental chlamydiae are a potential etiological agent of hospital-acquired pneumonia. Our recent study found that both DNAs of *Parachlamydia* and amoebae were coincidentally detected in a hospital environment, with the presence of *Acanthamoeba* having a significant effect on the long-term survival of the bacteria [9].

Thus, it is possible that this human pathogen can spread through a hospital environment via *Acanthamoeba*. It is important to determine whether this is the case for the control of hospital-acquired infection. However, whether amoebae harboring environmental chlamydiae can actually survive in harsh conditions, such as on floors or in sinks of hospitals, remains unknown. In the present study, we therefore isolated living amoebae containing symbiotic chlamydiae with bacterial pathogenic features from hospital environments.

## Methods

### Cells

*Acanthamoeba castellanii* C3 (ATCC 50739) were purchased from the American Type Culture Collection (ATCC). Amoebae were maintained in PYG broth (0.75% peptone, 0.75% yeast extract, and 1.5% glucose) at 30 °C [10]. *Parachlamydia* Bn_9_ (ATCC VR-1476) was also purchased from the ATCC, and the bacteria were propagated in an amoeba culture system according to methods described previously [10]. The numbers of bacterial infectious progeny were determined by the amoebal infectious unit (AIU) assay described previously [10]. The immortal human epithelial cell line, HEp-2, was also used for the study. HEp-2 cells were cultured in Dulbecco’s Modified Eagle’s Medium (DMEM, Sigma) containing 10% heat-inactivated fetal calf serum and antibiotics (penicillin, 100u/ml; streptomycin, 100 μg/ml) (Sigma) at 37 °C in 5% CO_2_.

### Smear sample collection

One hundred-smear samples were collected from a hospital (Hokkaido University Hospital, Sapporo, Japan, which has approximately 900 beds); 50 samples were collected in a winter trial, February to March 2012, and 50 samples were collected in a summer trial, August 2012. The smear samples were collected from the floor or sink outlet by wiping with sterilized gauze moistened with Page’s amoeba saline (PAS) [11], according to a previously described procedure [9]. The pellets obtained from the gauze were resuspended in PAS and used for amoebal isolation and DNA extraction. All sampling locations were limited at drainages, sinks and floors in the public space of hospital, which can be freely accessed by both patients and medical staffs, but not including emerging rooms with recovery rooms or patient rooms.

### Isolation of amoebae

Amoebae were isolated using a previously reported method [12]. In brief, a drop of sample/PAS solution was placed on the center of a non-nutrient agar plate on which heat-inactivated *E. coli* (a stock collection in our laboratory) were spread as a food source. Plates were then incubated at 30 °C. After 7 days of incubation, crawling amoebae with arm-like structures characteristic of *Acanthamoeba* cysts were isolated, according to a morphological assessment procedure [13]. Amoebae picked under microscopic observation from non-nutrient agar plates were then continuously grown in PYG broth to achieve axenic cultures. Three amoebal strains harboring environmental chlamydiae were finally established (Amoebal strain name/amoebal genotype/bacterial genus; W-9/T4/*Protochlamydia* sp., Y-20/T4/*Neochlamydia* sp., Y-23/T4/*Neochlamydia* sp.); however, because of lacking secondary infectious ability to C3 amoebae, Y-20 and Y-23 amoebae were omitted from the following experiments into assessing intracellular growth and IL-8 induction.

### Direct sequencing and phylogenic analysis

To identify *Acanthamoeba* and environmental chlamydiae in the isolates, total DNA was extracted from amoebae using a High Pure PCR Template Preparation Kit (Roche, Indianapolis, IN, USA), according to the manufacturer’s instructions. Extracted DNA was then amplified using High-Fidelity Phusion DNA polymerase (Thermo Fisher Scientific, San Jose, CA, USA) with specific primer sets for the *Acanthamoeba* 18S rRNA gene (JDP1, 5′-GGCCCAGATCGTTTACCGTGAA-3′; JDP2, 5′-TCTCACAAGCTGCTAGGGAG TCA-3′) [9] and the environmental chlamydia 16S rRNA gene (Ch5, 5′-CGTGGATGAGGCATGCRAGTCG-3′; Ch6, 5′-GTCATCRGCCYYACCTTVSR CRYYTCT-3′) [9]. The amplified products were separated by agarose gel electrophoresis and extracted from the gel using the FastGene Gel/PCR Extraction Kit (NIPPON Genetics, Tokyo, Japan) according to the manufacturer’s protocol, and then sequenced by Macrogen (Seoul, South Korea). A phylogenetic tree was constructed using the Neighbor-Joining method in MEGA software (version 4) [14]. Accession numbers of nucleotide sequences used for the phylogenetic analysis were listed into a table (See Additional file 1).

### Infection and bacterial detection

Bacteria (*Prochlamydia* W-9 or *Parachlamydia* Bn_9_) were added to each well of a 24-well plate seeded with C3 amoebae in PYG broth at a multiplicity of infection (MOI) of 1 and then incubated for 1 h. After washing, the cultures were further incubated for up to 5 days at 30 °C in a normal atmosphere. During this period, the amoebal cells were regularly collected to assess bacterial numbers using quantitative real-time PCR (qPCR), the AIU assay and DAPI staining, according to methods described previously [10, 15]. Meanwhile, the immortal human cell line, HEp-2, was also infected with the bacteria at a MOI of 1–5. The infected HEp-2 cells were incubated in DMEM containing 10% FCS with antibiotics for up to 5 days at 37 °C in a 5% CO_2_ atmosphere. Cells and supernatant were regularly collected for the determination of bacterial numbers and IL-8 secretion, respectively.

### IL-8 quantification

The amount of IL-8, which is an inflammatory cytokine, in HEp-2 cell culture supernatant was quantified using a commercial kit, Human IL-8 ELISA MAX™ Deluxe (BioLegend, Tokyo, Japan), according to the manufacturer’s protocol. The level of IL-8 gene expression was also determined by qRT-PCR using primer sets specific to IL-8 and an internal control (*gapdh*: glyceraldehyde-3-phosphate dehydrogenase) as described previously [16].

### Draft genome analysis and contig sequence accession numbers

*Protochlamydia* W-9 genomic DNA was prepared as described previously [17]. In brief, bacteria were collected from amoebae after disruption by bead-beating and were treated with DNase (Sigma) for 30 min at room temperature. After washing, the treated bacteria were suspended in 10 mM HEPES buffer containing 145 mM NaCl, and then the suspension was carefully overlayed onto 30% Percoll. The bacteria were collected from the lower layer following centrifugation at 30,000 × *g* for 30 min. Bacterial genomic DNA was extracted from the bacterial pellets with the High Pure PCR Template Preparation Kit as described above. The *Protochlamydia* W-9 draft genome was obtained using an Illumina Miseq sequencer (Illumina, San Diego, CA, USA), with sequencing runs for paired-end sequences. The bacterial DNA libraries were prepared using an NEBNext DNA Library Prep master mix set for Illumina (New England Biolabs, Ipswich, MA, USA). The genome was assembled using *de novo* sequence assembler software (Platanus 1.2.1) [18]. Rapid Annotation using Subsystem Technology (RAST: http://rast.nmpdr.org/) was used for gene annotation [19]. Also, functional annotation was performed with the Kyoto Encyclopedia of Genes and Genomes (KEGG) (http://www.genome.jp/kegg/) [20]. The draft genome sequence of *Protochlamydia* W-9 has been deposited in the DDBJ database under accession numbers BCPZ01000001-BCPZ01000402 (402 entries) and Bioproject number: PRJDB4526.

### Statistical analysis

Data were compared using Student’s *t*-test. Also, prevalence between trials was compared using Pearson’s chi-square test. A *p*-value of less than 0.05 was considered significant.

## Results

### Prevalence of living amoebae captured in hospital environments and establishment of an amoebal strain harboring an environmental chlamydiae

**Fig. 1:**
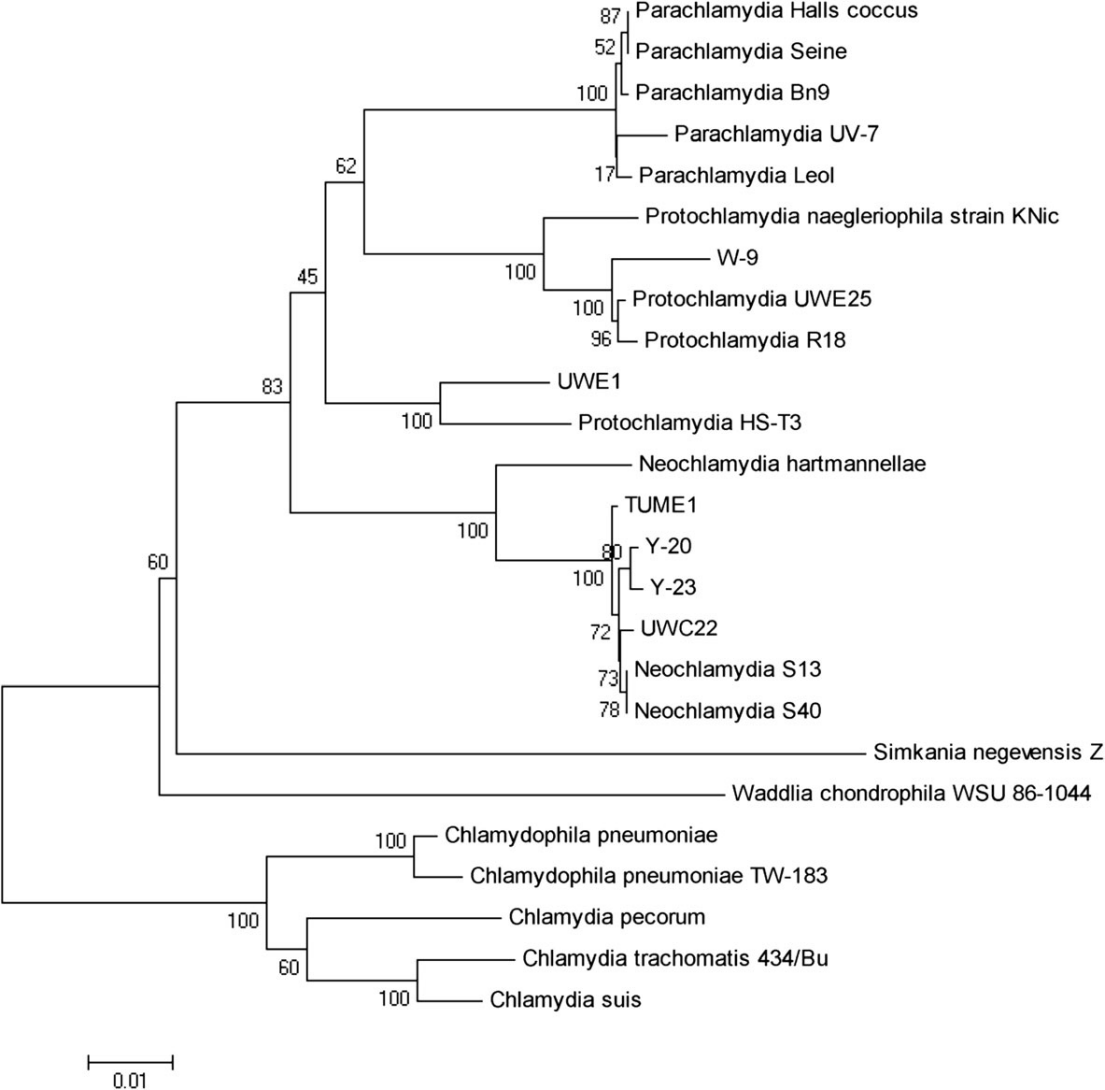
Phylogenetic tree for *Protochlamydia* W-9 and other chlamydiae 16S rRNA gene sequences. The tree was constructed by using the Neighbor-Joining method in MEGA software (version 4) [14]. Accession numbers (nucleotide sequences) used for phylogenetic analysis are listed in the Material and Methods section

From 100 swabs taken from hospital floors and sinks, 23 amoebae were enriched on agar plates spread with heat-killed *E. coli*. From these, eleven were successfully cultured in PYG medium (Additional file 2). Prevalence appeared to increase in the Summer trial, but without statistical significance (*p* = 0.096), potentially indicating a seasonal variation. Meanwhile, there was no difference in prevalence between swabs from either ‘Dry’ or ‘Moist’ conditions or between floors. One amoeba strain (W-9 amoebae) with an environmental chlamydia was finally used for the following studies; because of lacking secondary infectious ability to C3 amoebae, Y-20 and Y-23 amoebae were omitted from the following experiments into assessing intracellular growth and IL-8 induction. Phylogenic analysis revealed that the W-9 amoeba and the endosymbiotic chlamydia were *Acanthamoeba* genotype T4 (Additional file 3) and *Protochlamydia* sp. (Fig. 1), respectively. Thus, living amoebae are distributed in hospital environments and occasionally contain environmental chlamydiae.

### *Protochlamydia* W-9 intracellular growth and IL-8 induction

**Fig. 2:**
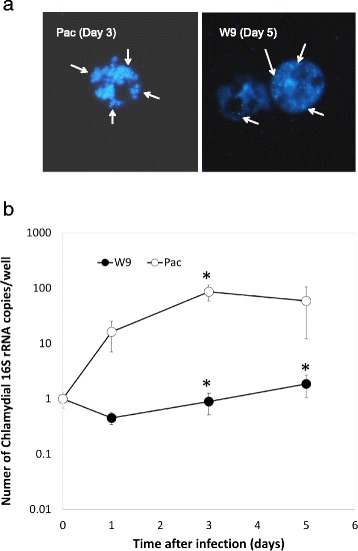
Growth of *Protochlamydia* W-9 (W9) and *Parachlamydia* Bn_9_ (Pac) in *Acanthamoeba* C3. **a** Representative images of DAPI staining showing *Protochlamydia* W9-infected C3 amoebae (Day 5 post-infection) and *Parachlamydia* Bn_9_-infected C3 amoebae (Day 3 post-infection). Arrows show bacterial clusters in each amoeba. **b** Changes in the number of chlamydial 16S rRNA copies per well. The data represent the average number of copies ± SD. The bacterial 16S rRNA copy numbers were normalized with the amoebal 18S rRNA copy numbers. *indicates *p* < 0.05 vs. value at 1 day

**Fig. 3:**
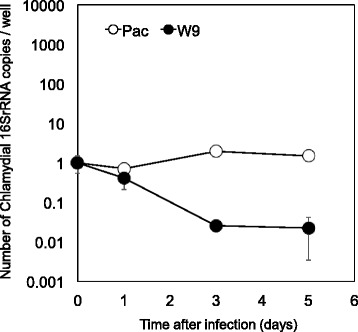
Growth of *Protochlamydia* W-9 (W9) and *Parachlamydia* Bn_9_ (Pac) in the immortal human epithelial cell line, HEp-2. The data show changes in the average number of chlamydial 16S rRNA copies per well, ± SD. The bacterial 16S rRNA copy numbers were normalized with the HEp-2 *gapdh* copy numbers

**Fig. 4:**
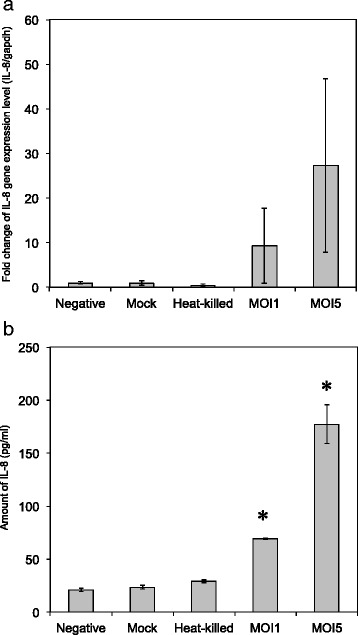
IL-8 induction in *Protochlamydia* W-9-stimulated HEp-2 cell cultures. **a** Assessment of IL-8 gene expression in cultures (24 h post-infection). The level of IL-8 gene expression was determined according to qPCR using primer sets specific to IL-8 and an internal control (GAPDH: glyceraldehyde-3-phosphate dehydrogenase). The data represent the average copies ± SD. **b** Amounts of IL-8 in culture supernatants. The amounts of IL-8 were quantified with a commercial kit, Human IL-8 ELISA MAX™ Deluxe. The data represent the average amount ± SD (pg/ml). * indicates *p* < 0.05

We next assessed if the *Protochlamydia* W-9 could secondarily infect and then grow in C3 amoebae. qRT-PCR assessment revealed an increase in the amount of bacterial DNA (W-9) in cultures depending on culture period, although the growth speed was very slow compared with that of *Parachlamydia* Bn_9_ (Pac) (Fig. 2b). DAPI staining showed a few bacterial particles; however, *Parachlamydia* Bn_9_-infected amoebae were mostly disrupted at 3 days (Fig. 2a), supporting our hypothesis that *Protochlamydia* W-9 grows slowly in the host amoebae. In contrast to C3 amoebae, the *Protochlamydia* W-9 failed to grow in HEp-2 cells with bacterial numbers decreasing rapidly below baseline after 1 day of incubation (Fig. 3). Interestingly, both ELISA and qRT-PCR revealed that stimulation with living bacteria, but not with heat-killed bacteria, significantly induced IL-8 secretion from HEp-2 cells (Fig. 4). Taken together, these results indicate that *Protochlamydia* W-9 could spread easily in hospital environments through amoebal secondary infection, supporting our previous results [9]. Furthermore, because of the induction of IL-8, the bacteria could exacerbate inflammation, potentially indicating a risk of a hospital-acquired infection.

### The *Protochlamydia* W-9 draft genome features several genes associated with resistance to antibiotics and toxic compounds

The draft genome of *Protochlamydia* consisted of 2,484,573 bp (total contig size) in 402 scaffold contigs. The genome contains 2100 protein-coding sequences (CDSs), and 49 RNAs. RAST-annotated features revealed that there were several genes associated with resistance to antibiotics and toxic compounds, indicating the ability of the bacteria to adapt to hash environments (Table 1). Comparative genome analysis with KEGG revealed the *Protochlamydia* W-9 genome possessed representative environmental chlamydial metabolic pathways (present in *Protochlamydia* UWE25 [2] and *Protochlamydia* R18 [21]) that are required for amoebal adaptation (Additional file 4); however, it lacked the genes encoding the type IV secretion system (Additional file 5).

**Table 1:** RAST- annotated genes associating with resistance to antibiotics and toxic compounds

RAST Subsystem	Role (KEGG located number)	Features
Copper homeostasis	Copper-translocating P-type ATPase (EC 3.6.3.4)	peg.608, peg.990, peg.1254
Cobalt-zinc-cadmium resistance	Heavy metal RND efflux outer membrane protein, CzcC family	peg.605
	Cobalt-zinc-cadmium resistance protein	peg.1410
	Cobalt/zinc/cadmium efflux RND transporter, membrane fusion protein, CzcB family	peg.606
	Cobalt-zinc-cadmium resistance protein CzcA	peg.607
	Cation efflux system protein CusA	peg.607
Resistance to fluoroquinolones	DNA gyrase subunit B (EC 5.99.1.3)	peg.2036
	DNA gyrase subunit A (EC 5.99.1.3)	peg.2037
	Topoisomerase IV subunit B (EC 5.99.1.-)	peg.888
	Topoisomerase IV subunit A (EC 5.99.1.-)	peg.889
Beta-lactamase	Beta-lactamase (EC 3.5.2.6)	peg.1027, peg.1665
	Metal-dependent hydrolases of the beta-lactamase superfamily I	peg.123
Multidrug Resistance Efflux Pumps	RND efflux system, outer membrane lipoprotein, NodT family	peg.1170
	RND efflux system, membrane fusion protein CmeA	peg.1631
	RND efflux system, outer membrane lipoprotein CmeC	peg.1629
	Acriflavin resistance protein	peg.521
	Membrane fusion protein of RND family multidrug efflux pump	peg.522
	RND efflux system, inner membrane transporter CmeB	peg.1630
Multidrug Resistance Efflux Pumps	Multidrug and toxin extrusion (MATE) family efflux pump YdhE/NorM, homolog	peg.1277

## Discussion

We have previously reported both *Acanthamoeba* and environmental Chlamydia (*Parachlamydia*) DNAs in a hospital environment, and have shown in an in vitro study that the presence of *Acanthamoeba* has a significant impact on the long-term survival of environmental chlamydiae. These findings raise the possibility that this potential human pathogen could spread through a hospital environment via *Acanthamoeba* [9]. However, whether amoebae harboring environmental chlamydiae actually inhabit hospital environments remained to be clarified. We therefore attempted to isolate living amoebae with symbiotic chlamydiae from hospital environments.

From 100 samples obtained from floors or sinks, 23 amoebae were enriched on agar plates spread with heat-killed *E. coli*. Consistent with PCR results, 21 of these amoebae had morphological features identical to those of *Acanthamoeba* cysts, such as arm-like structures [13] (See Additional file 2). These results indicated that the amoebal enrichment method was very accurate with high sensitively, despite being time consuming. The amoebae were isolated from places such as floors or sinks in the hospital, suggesting that amoebae inhabit hospital environments. However, because prevalence in the summer tended to be higher than that in the winter, it is possible that outpatients carry less amoeba-containing soil into the hospital during winter because of snow. Chlamydial isolates (Y-20, Y-23 and W-9) have come from distinct 3 floors (7, 8 and 10)(See Additional file 1) directly connected by an elevator, which may be a potential factor responsible for reflecting bacterial traffic via human flow into public spaces. It appeared that there were more amoeba isolated on the lower floors compared to the higher ones, although sample numbers were different among floors, supporting the scenario. Further studies with other hospitals should be performed to clarify this possible scenario. Half of the amoebae enriched on agar plates with heat-killed bacteria (a conventional isolation procedure) failed to grow in PYG medium cultures. The procedure for amoebal isolation using PYG axenic culture may preferentially select some amoebae, indicating a limitation of this protocol. Furthermore, only one amoebal strain with an endosymbiont (*Protochlamydia* W-9) was fully used for assessing intracellular growth and IL-8 induction, because of the other amoebal endosymbionts (*Neochlamydia* spp.) lacking secondary infectious ability to C3 amoebae. Although the exact reason for the lacking ability remains to be clear, the findings were intriguingly identical to our previous studies [12, 21], indicating a wide range of diversity in environmental chlamydiae. Thus, the results reveal the presence of a complicated and unknown amoebal ecology in hospital environments, as well as natural environments.

As mentioned above, the growth of *Protochlamydia* W-9 was very slow compared with the other chlamydia (*Parachlamydi*a Bn_9_), which caused rapid amoebal lysis depending on bacterial maturation (See Fig. 2). Several reports reveal that amoebae inhabit a wide range of environments depending upon food sources, raising the idea that the amount of food on artificial surfaces, such floors or sinks, might be less than that in natural environments, such as soil or pond waters [12, 22–24]. It is, therefore, possible that slow bacterial growth can be beneficial for saving food in harsh environments. Meanwhile, the bacteria failed to grow in a representative immortal human epithelial cell line, HEp-2. Surprisingly, the amount of bacteria significantly decreased during the culture period compared to a reference strain, *Parachlamydia* Bn_9_ [25]. While these findings indicate a rapid elimination of bacteria from the cells, it raises a possible scenario that the host cells can rapidly sense the bacteria, which stimulates pro-inflammatory cytokines.

Draft genome analysis revealed *Protochlamydia* W-9 to possess principal metabolic features that are common to environmental chlamydiae, such as *Protochlamydia* UWE25 or R18. Also, our data interestingly showed the *Protochlamydia* W-9 genome to contain several genes associated with resistance to antibiotics and toxic compounds, although this feature was not unique to *Protochlamydia* W-9 because it was seen in other bacterial genomes (data not shown). Accumulated evidence indicates that predatory amoebae play a role in transferring bacterial gene fragments from bacterial pray to other bacteria [26–28]. Indeed, protozoa (such as ciliates) are potentially a device for the acceleration of bacterial horizontal gene transfer [29–31]. Thus, amoebae harboring environmental chlamydiae with such resistance genes or detoxification systems may be a source of these genes for other inhabitants of amoebae, although further study is needed to test this hypothesis. Meanwhile, when compared with the other bacteria (UWE25 and R18), the type IV secretion system, which delivers effector proteins associated with host cell modulation [32], was not seen, indicating a divergent evolution of environmental chlamydiae. Meanwhile, because environmental chlamydiae (*Parachlamydia*) lacking the type IV secretion system replicated in immortal epithelial cells, albeit not at body temperature [25], *Protochlamydia* W-9 has the potential to adapt to human cells.

## Conclusions

We visualized for the first time living *Acanthamoeba* in hospital environments, identifying a risk of environmental chlamydiae, *Protochlamydia* W-9, in evoking inflammation. Although further study with extended sampling is needed to confirm our findings, our results are useful for understanding amoebal ecology in complicated hospital environments, and indicate a potential role of environmental chlamydiae in evoking respiratory inflammation through inhalation.

## Additional files

Additional file 1: Accession numbers of nucleotide sequences used for the phylogenetic analysis. (XLSX 21 kb)
Additional file 2: Prevalence of amoebae with or without environmental chlamydiae in the hospital. (XLSX 34 kb)
Additional file 3: Phylogenetic tree for Acanthamoeba 18S rRNA with W-9 toY-23 amoebae. The tree was constructed by using the Neighbor-Joining method in MEGA software (version 4). Nucleotide sequences (Accession numbers) used for the phylogenetic analysis were listed in the Material and Methods section. (PPTX 285 kb)
Additional file 4: Comparison of metabolic pathways between *Protochlamydia* W-9 (BCPZ01000001-BCPZ01000402) and a representative chlamydiae (*Protochlamydia* UWE25 [2] and *Protochlamydia* R18 [20]). Green lines, unique in the *Protochlamydia* W-9 active modules. Blue lines, shared modules. Red lines; modules specific for *Protochlamydia* UWE25. (PPTX 496 kb)
Additional file 5: Comparative genomic features of *Protochlamydia* W-9 (BCPZ01000001-BCPZ01000402) and *Protochlamydia* R18 [20] aligned on a representative chlamydiae, *Protochlamydia* UWE25 [2]. Cicles 1 and 2 show the aligned genomic identity of *Protochlamydia* W-9 and *Protochlamydia* R18, respectively. * indicates lacking the genes encoding type IV secretion system. (PPTX 438 kb)

## Abbreviations

ELISA: Enzyme-linked Immuno-sorbent assay
