# Supplementary figures and images for: Acanthamoeba containing endosymbiotic chlamydia isolated from hospital environments and its potential role in inflammatory exacerbation
Source: BMC Microbiol. 2016 Dec 15;16:292. doi: 10.1186/s12866-016-0906-1 (PMC5160005; doi:10.1186/s12866-016-0906-1)

## Slide 1
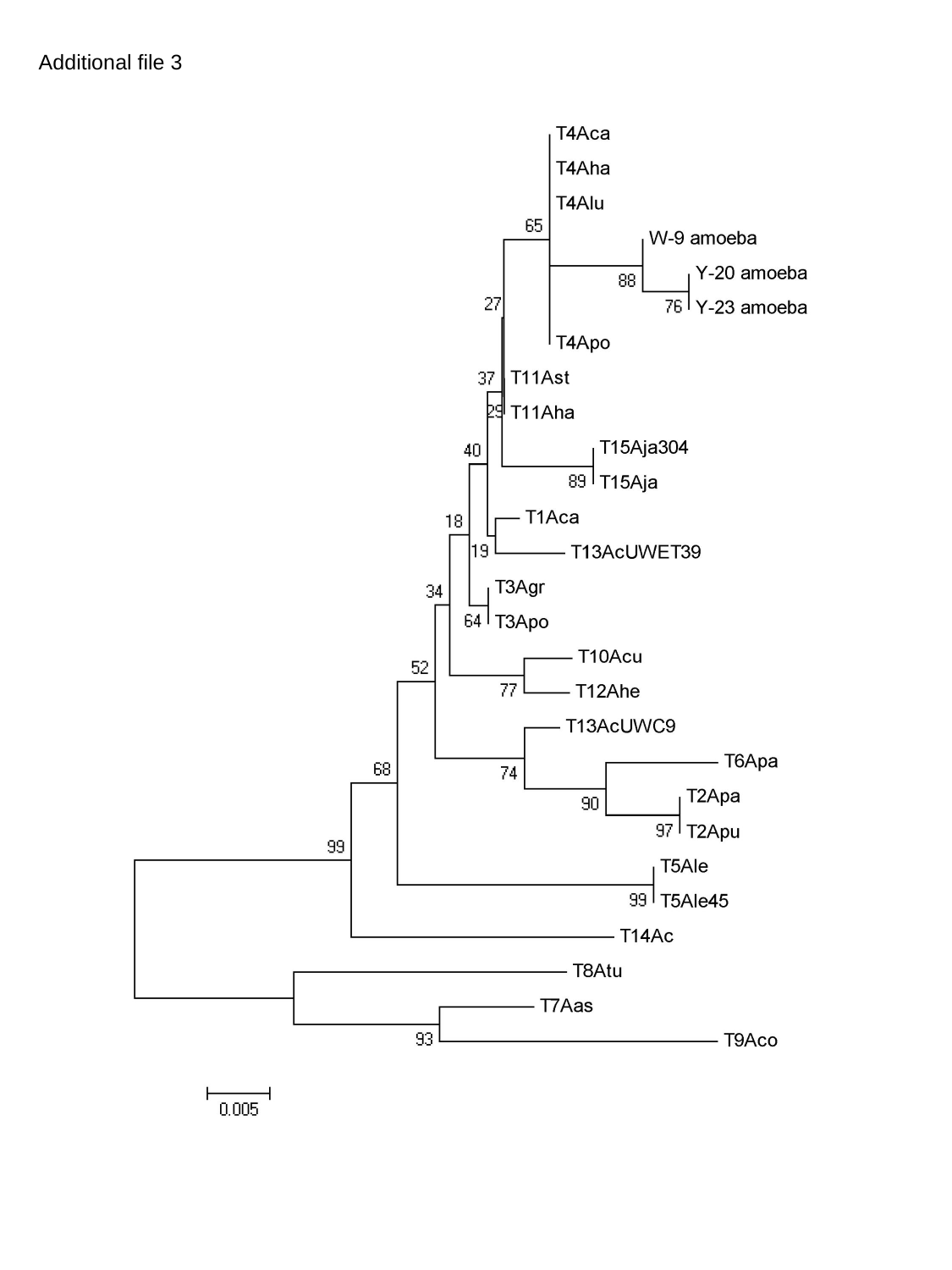

Additional file 3

Supplement: Additional file 3: — Phylogenetic tree for Acanthamoeba 18S rRNA with W-9 toY-23 amoebae. The tree was constructed by using the Neighbor-Joining method in MEGA software (version 4). Nucleotide sequences (Accession numbers) used for the phylogenetic analysis were listed in the Material and Methods section. (PPTX 285 kb) [file 12866_2016_906_MOESM3_ESM.pptx]

## Slide 1
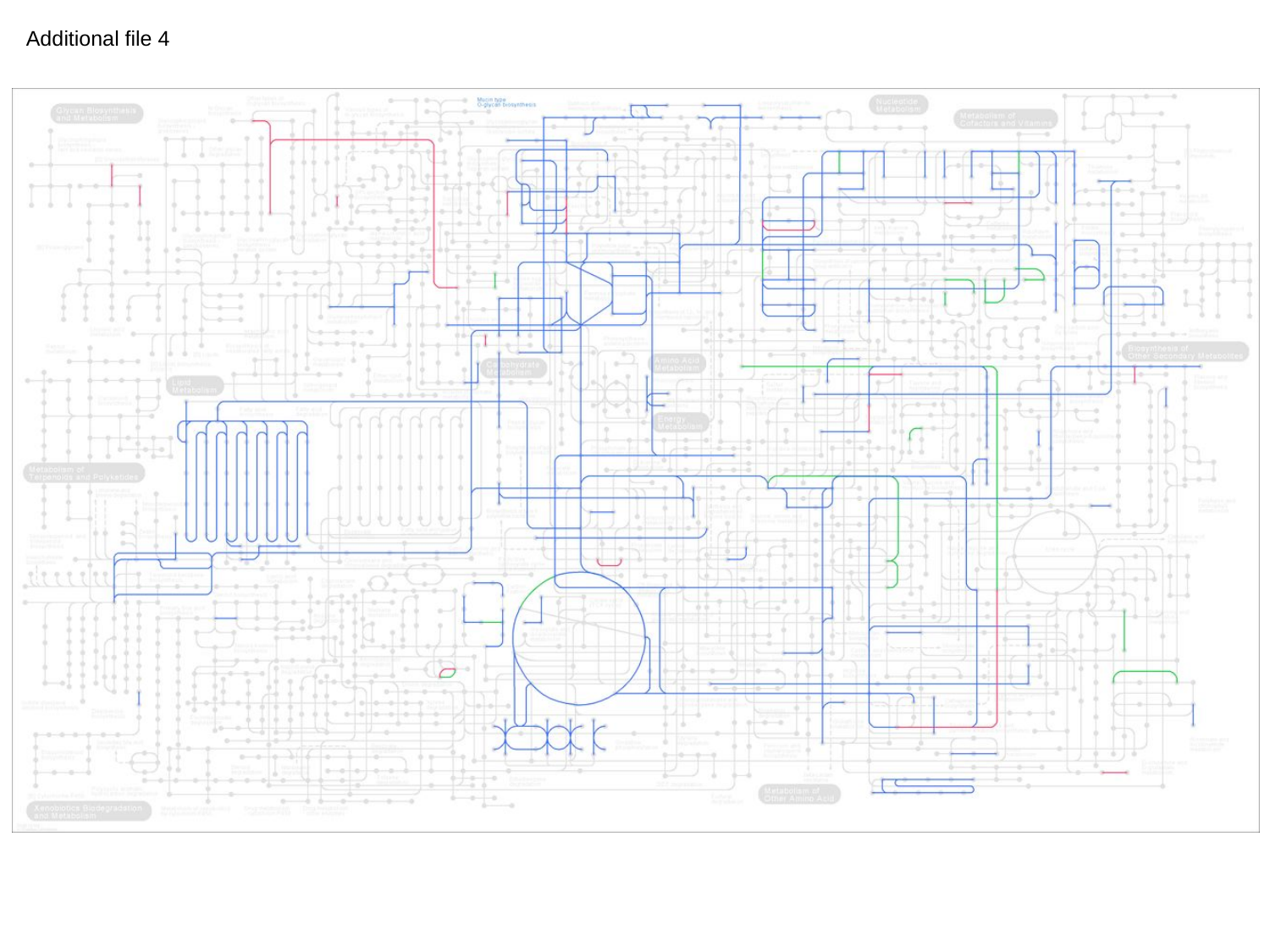

Additional file 4

Supplement: Additional file 4: — Comparison of metabolic pathways between Protochlamydia W-9 (BCPZ01000001-BCPZ01000402) and a representative chlamydiae (Protochlamydia UWE25 [2] and Protochlamydia R18 [20]). Green lines, unique in the Protochlamydia W-9 active modules. Blue lines, shared modules. Red lines; modules specific for Protochlamydia UWE25. (PPTX 496 kb) [file 12866_2016_906_MOESM4_ESM.pptx]
