# Supplementary material for: Acanthamoeba containing endosymbiotic chlamydia isolated from hospital environments and its potential role in inflammatory exacerbation
Source: BMC Microbiol. 2016 Dec 15;16:292. doi: 10.1186/s12866-016-0906-1 (PMC5160005; doi:10.1186/s12866-016-0906-1)
Supplement: Additional file 5: — Comparative genomic features of Protochlamydia W-9 (BCPZ01000001-BCPZ01000402) and Protochlamydia R18 [20] aligned on a representative chlamydiae, Protochlamydia UWE25 [2]. Cicles 1 and 2 show the aligned genomic identity of Protochlamydia W-9 and Protochlamydia R18, respectively. * indicates lacking the genes encoding type IV secretion system. (PPTX 438 kb) [file 12866_2016_906_MOESM5_ESM.pptx]

## Slide 1
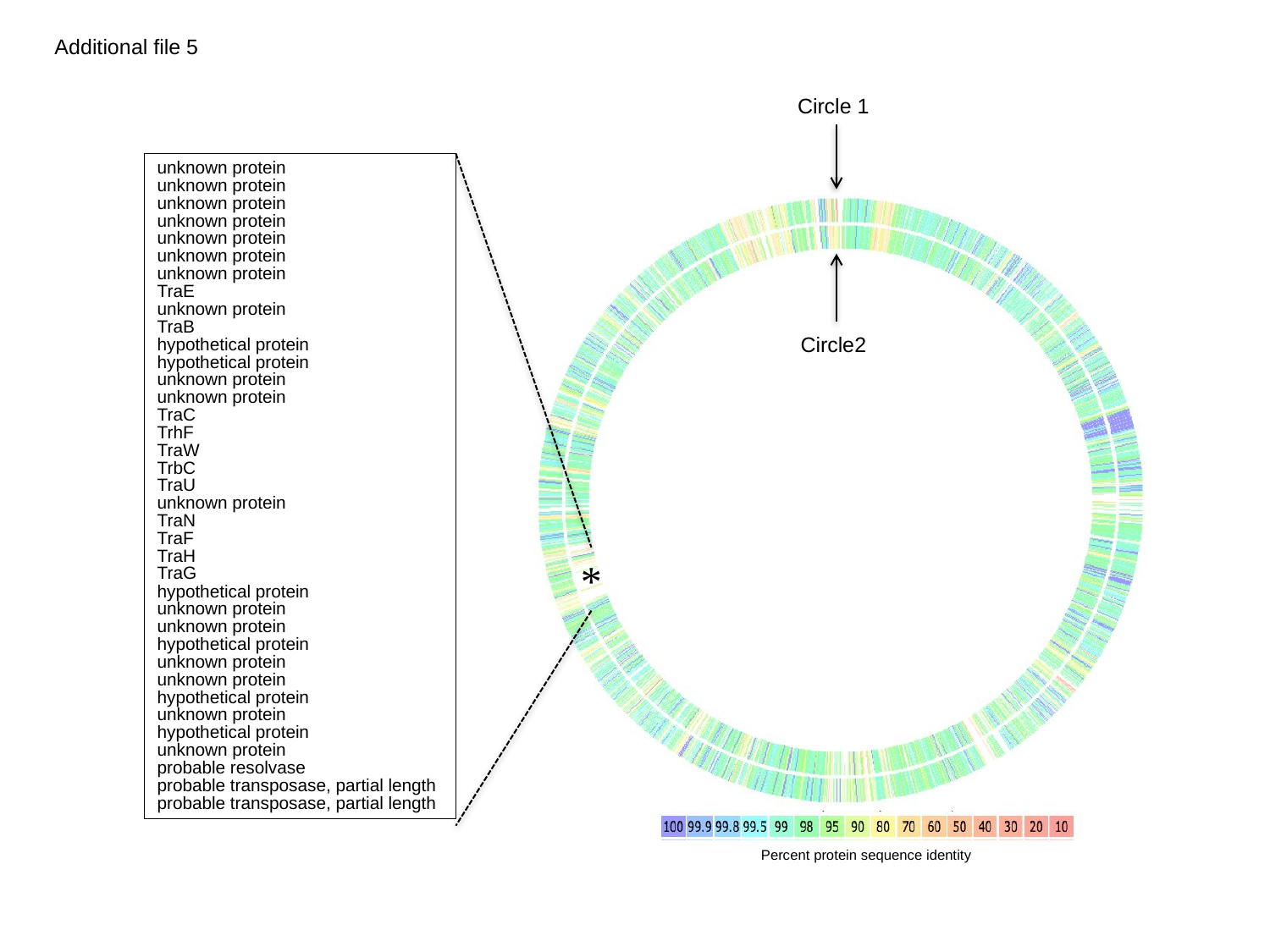

Additional file 5
Circle 1
unknown protein
unknown protein
unknown protein
unknown protein
unknown protein
unknown protein
unknown protein
TraE
unknown protein
TraB
hypothetical protein
hypothetical protein
unknown protein
unknown protein
TraC
TrhF
TraW
TrbC
TraU
unknown protein
TraN
TraF
TraH
TraG
hypothetical protein
unknown protein
unknown protein
hypothetical protein
unknown protein
unknown protein
hypothetical protein
unknown protein
hypothetical protein
unknown protein
probable resolvase
probable transposase, partial length
probable transposase, partial length
Circle2
*
Percent protein sequence identity
